# The potential of unstructured “physical activity” data for understanding relationships between movement-induced joint loading and osteoarthritis progression

**DOI:** 10.1016/j.ocarto.2025.100731

**Published:** 2025-12-12

**Authors:** Peter Schaefer, Zoe Struk, Kerry E. Costello

**Affiliations:** Mechanical & Aerospace Engineering, University of Florida, FL, USA

**Keywords:** Wearable sensors, Biomechanics, Real-world movement, Activity variability, Data science, Digital health

## Abstract

Gait and exercise have been extensively studied in knee osteoarthritis (OA) as potential interventions to modify mechanical loading at the joint and, subsequently, influence biological processes and disease progression. However, this research has often failed to account for mechanical loading encountered in daily life outside structured activities. Wearable sensors help address this limitation by capturing movement as it occurs in daily life. Yet most analyses have relied on coarse summary measures (e.g., step count), overlooking biologically relevant variation in loading patterns across activities and time. Given that these sensors record millions of data points per day, there is an opportunity to move beyond summary measures and quantify within- and between-day variations in movement patterns. We propose that a deeper exploration of these rich datasets, guided by OA literature and related fields, may reveal how load-inducing human movement contributes to knee OA, informing the development of personalized, activity-based interventions.

## 1 Introduction

Mechanical loading on the knee is one of the few readily modifiable risk factors for knee osteoarthritis (OA), making it a valuable target for interventions. This has led to decades of research on gait retraining and exercise programs as strategies to influence movement and, in turn, the mechanical loading experienced by the knee. However, the effects of these programs have been modest at best [1], perhaps because the ways in which humans accumulate physical activity are not limited to walking or structured exercise alone. Pathological responses of joint tissues depend on the specific temporal patterns of loading experienced (i.e., magnitudes, durations, and frequencies). Therefore, characterizing how unstructured activity is accumulated in daily life may provide greater insight into how movement-induced joint loading affects knee OA.

## 2 Expanding beyond typical and planned activity

Laboratory-based gait analysis quantifies joint loading during a “typical” step of one of the most common daily activities, but this highly controlled setting does not fully capture how people actually move in the real world [2]. In daily life, load-inducing activities are more varied, and movement patterns shift with contextual factors such as environmental conditions (e.g., terrain, lighting), external demands (e.g., traffic, cell phone use), and physical or psychological states (e.g., pain variability, depression) [3,4]. Recently reported within-day gait variability in individuals with knee OA [5] further highlights the disconnect between “typical” lab data and real-world movement. This challenge was recognized over 15 years ago, when Maly introduced a cumulative load framework combining daily activity volume with lab-measured joint loading [6]. However, this framework still relies on a single “typical” movement pattern, even though an individual can accomplish the same activity using different movement patterns, resulting in different joint loading profiles even for the same task. Thus, while Maly's work addressed an important gap, alternate methods are needed to bridge this disconnect, capture the variability of load-inducing movement patterns outside the lab, and ultimately support more effective biomechanical interventions.

Exercise programs, despite being one of the few strongly recommended non-pharmaceutical treatments for managing knee OA [7], face a similar challenge. Meta-analyses show short-term symptom improvements with exercise [8], yet these benefits are often modest and tend to diminish over time. Moreover, because exercise emphasizes planned, structured activities, the contribution of unstructured daily activity (e.g., light-intensity walking, household tasks) to joint loading and potential OA progression is often overlooked [9,10]. Failing to account for this full spectrum of physical activity provides an incomplete picture of the potential activity-induced biological responses in joint tissues.

Thus, while gait interventions and exercise programs provide important starting points, accounting for the inherent variability of unstructured daily movement may be necessary to achieve more favorable treatment outcomes.

## 3 Expanding beyond summary measures

Wearable inertial measurement units (IMUs) have emerged as a dominant method for capturing detailed movement data outside laboratory settings, offering new opportunities to understand real-world activity and its impact on joint health. These compact, inexpensive devices typically record tri-axial acceleration (sometimes with angular velocity and heading information) at 30–100 Hz, generating millions of data points per device per day. Although not direct measures of mechanical loading, these high-frequency signals may capture variability in load-inducing activity relevant to OA progression. Despite this potential, most knee OA studies still rely on summary measures such as average daily step count, sacrificing information about temporal structure in load-inducing activity that may be contained within these rich signals. Other common summary measures, such as minutes of moderate-to-vigorous physical activity (MVPA), reflect overall activity intensity but still obscure the biologically meaningful temporal structure of load-inducing movement. Moving beyond summary measures to analyze the rich temporal structure of raw sensor data may reveal patterns of load-inducing activity that influence pathological responses and long-term joint health.

While relationships between activity accumulation and knee OA outcomes are not yet established, work in other fields demonstrates that high-frequency wearable sensor data contain meaningful temporal patterns. In circadian research, accelerometer-derived rest-activity rhythms, which describe how activity is distributed and repeated across days, distinguish healthy from pathological aging [11,12]. Applying similar approaches in OA could provide a holistic view of real-world activity variation and identify novel activity-based intervention targets. In parallel, advances in human activity recognition have emerged from analyzing intrinsic properties of the IMU signal (e.g., range, variability, frequency composition) across sub-daily timescales [13]. Adopting this signal-centric approach in OA may provide a foundation for characterizing unstructured daily movement and reveal distinctive load-inducing movement patterns associated with OA pathology. High-frequency signals may also capture instances of high-magnitude acceleration or angular velocity, allowing potential quantification of microtraumas such as “stepping funny and tweaking the knee.” Exploring how these temporal signal features relate to OA progression may clarify how unstructured, real-world movement influences joint health and inform targeted, activity-based interventions.

## 4 Clinical applications

Through the commercial success of devices like Apple Watches and Fitbits, wearable sensors have become ubiquitous, presenting new opportunities for activity-based knee OA management. Although physical activity is widely recommended as a therapeutic tool, patients often receive limited guidance on the specific types, intensities, or timing of activities they should engage in or avoid. This gap is reflected in health care counseling trends: across arthritic conditions more broadly, fewer than 20 % of adults report receiving physical activity counseling in the past six months, and only 4.4 % receive a recommendation for arthritis-appropriate activity [14]. This limited support may contribute to increased sedentary time, as patients experiencing pain may hesitate to engage in activity without reassurance or clear guidance [15]. Wearable sensing offers a potential solution by capturing real-world movement patterns, enabling more precise, data-driven activity recommendations along with opportunities for daily monitoring and just-in-time interventions delivered through digital health platforms.

## 5 Remaining challenges to high resolution activity investigation

Patient wear compliance, battery life, data storage, and data complexity remain key considerations before high-frequency IMU data can be effectively used to understand the relationship between activity patterns and knee OA. Wrist- and low-back-worn devices facilitate compliance but capture arm or whole-body movement rather than knee-specific movement, limiting potential for direct translation to joint loading. Multi-sensor approaches can characterize movement patterns during real-world activity, but their complexity and wear burden currently limit feasibility for sustained, everyday use or integration into digital health monitoring tools.

High-frequency data also present challenges in device capability and data processing. Limited on-board storage and battery life constrain recording duration, particularly when angular velocity (gyroscope) data are collected, creating trade-offs between sampling frequency, signal type, and wear duration. The sheer volume of data complicates feature extraction, often requiring substantial computing resources.

Further work is needed to optimize data collection strategies, establish standardized pipelines for processing high-frequency sensor data, and, critically, identify which temporal signal features are most relevant for capturing meaningful variation in load-inducing movement. These efforts will also improve comparability across studies and clarify how real-world movement patterns can be leveraged to inform biomechanics and activity-based interventions for knee OA.

## 6 Conclusion

Exploring high resolution data from wearable sensors may allow for expansion beyond traditional, structured, and summary measures of movement to understand how the accumulation of load-inducing daily activity is related to knee OA progression. Although the optimal approaches for analyzing and interpreting these large datasets are still being established, advances in computational power, data science, and cross-disciplinary approaches offer new opportunities to extract meaningful information from these rich data sources. These insights could ultimately support development of just-in-time, patient-specific activity recommendations to improve OA care.

## Contributions

All authors contributed to conception and design, drafting of the article, critical revision of the article for important intellectual content, and final approval of the article. KEC, on behalf of all authors, takes responsibility for the integrity of the work as a whole.

## Declaration of generative AI and AI-assisted technologies in the writing process

During the preparation of this work the authors used ChatGPT-5 (OpenAI) in order to lightly revise the text for length and clarity. After using this tool/service, the authors reviewed and edited the content as needed and take full responsibility for the content of the publication.

## Role of the funding source

Funding for this project was provided by the Rheumatology Research Foundation (Investigator Award, KEC). ZS was supported by an AI Scholars award from the University of Florida. The study sponsors were not involved in drafting the manuscript or the decision to submit the manuscript for publication.

## Declaration of competing interest

All authors have no conflicts of interest to report.
